# Mapping evidence for health policy and systems decision-making: a spectrum approach bridging tacit and scientific knowledge across local and global contexts

**DOI:** 10.1186/s12961-026-01502-4

**Published:** 2026-06-30

**Authors:** D. Waithaka, B. Tsofa, C. Glenton, J. Nzinga, A. Koduah, E. Barasa, S. Lewin, U. Gopinathan

**Affiliations:** 1 Health Economics Research Unit, KEMRI Wellcome Trust Research Programme, Nairobi, Kenya; 2 KEMRI Centre for Geographic Medicine Research, KEMRI Wellcome Trust Research Programme, Kilifi, Kenya; 3 Department of Health and Functioning, Western Norway University of Applied Sciences, Bergen, Norway; 4 Liverpool School of Tropical Medicine, Liverpool, United Kingdom; 5 Department of Pharmacy Practice and Clinical Pharmacy, School of Pharmacy, College of Health Sciences, University of Ghana, Accra, Ghana; 6 Centre for Global Health and Tropical Medicine, Nuffield Department of Medicine, University of Oxford, Oxford, United Kingdom; 7 Department of Health Sciences Ålesund, Norwegian University of Science and Technology (NTNU), Ålesund, Norway; 8 Centre for Epidemic Interventions Research, Norwegian Institute of Public Health, Oslo, Norway; 9 Health Systems Research Unit, South African Medical Research Council, Cape Town, South Africa; 10 Global Health Cluster, Division for Health Services, Norwegian Institute of Public Health, Oslo, Norway

**Keywords:** Evidence-informed policy, Decision-making approach, Evidence mapping, Policy analysis

## Abstract

**Background:**

Research on evidence-informed decision-making has commonly focused on scientific evidence. However, this does not reflect the diversity of evidence used in real-world policy settings or give decision-makers a clear way to characterize and compare different evidence sources. This article describes the development of a spectrum approach to map the types of evidence used in health policy decisions and support more systematic and transparent judgements about their nature and applicability to different decisions.

**Methods:**

The approach was developed in four stages. First, we conducted a targeted literature search to identify key papers defining the concept of “evidence”. From these, we initially categorized evidence as binary according to its nature (either tacit or scientific) and its geographic scope (either global or local). Second, we tested these categorizations using findings from a global systematic review and an empirical study on vaccine policy-making in Kenya. This showed that binary categorizations were inadequate for capturing the range of evidence informing decisions and transparently presenting how evidence sources vary in their generation and contextual applicability. Third, iterative team deliberations about these limitations led us to develop a spectrum approach that maps evidence along two intersecting axes: tacit–scientific and local–global. Finally, stakeholders provided feedback on the clarity, relevance and applicability of this approach.

**Results:**

The spectrum approach positions evidence along two axes: tacit to scientific (the extent to which evidence is independent of individual experience, documented and generated through systematic, transparent and reproducible processes) and global to local (in relation to the decision setting). Positioning evidence along axes rather than in binary categories allowed us to distinguish evidence that varies along these dimensions, visualize the forms of global and local evidence available and where gaps exist, and reflect more explicitly on the applicability of different evidence sources to specific decision contexts.

**Conclusions:**

Binary categorizations inadequately reflect variation in how evidence is generated and what evidence can be useful for decision-making. By mapping evidence across intersecting tacit–scientific and global–local continua, the spectrum approach offers a clear and inclusive framework that can support researchers and decision-makers in drawing more fully on the breadth of evidence available to inform health systems decisions.

**Supplementary Information:**

The online version contains Supplementary Material available at https://doi.org/10.1186/s12961-026-01502-4.

## Background

In recent decades, there has been a growing emphasis on the use of evidence to inform health policy and systems decision-making, grounded in the assumption that evidence-informed policies are more likely to be effective, efficient, equitable and/or accountable [1–6]. Yet, what constitutes evidence and how it should be used remain subjects of ongoing debates shaped by disciplinary traditions and contextual realities [7–11]. These debates matter in practice because decision-makers must often identify, assess and combine different forms of evidence when making policy choices [12, 13].

On the supply side, research environments have traditionally emphasized the generation and use of scientifically rigorous evidence [8, 11, 14–16]. Such evidence is generated through systematic, transparent and replicable methodologies, including primary research conforming to explicit reporting standards and systematic reviews. Within many of these environments, including health and medical research, the emphasis on global generalizability has left less room for generating, appraising and using locally grounded, experiential and practice-based evidence [4, 6, 11, 17–19]. However, research approaches have expanded in recent years. In health systems research, for example, there is growing acknowledgement of qualitative research, which focuses less on generalizability [20–22]. In addition, recent work has acknowledged the need to include what is described as unconventional sources of evidence into decision-making processes [4, 11, 23, 24]. This includes so-called tacit evidence that is not produced through formal scientific methods but is grounded in individual experiences or practices, often conveyed through personal interactions rather than formal documentation, and can include stakeholders’ opinions, values and practices [5]. Despite this interest in tacit and other unconventional sources of evidence, frameworks for systematically mapping and integrating these alternative forms of evidence remain underdeveloped, limiting how such evidence is described and considered alongside scientific evidence in decision-making.

On the demand side, policy-makers and practitioners often grapple with assessing whether evidence generated in other contexts (e.g. regionally or at the global level) can be applied meaningfully within their own settings [6, 25–28]. Additionally, they must also weigh the perceived credibility and contextual relevance of such evidence alongside locally competing priorities, stakeholder interests, resource constraints and institutional dynamics [2, 6, 29–35]. This can be challenging because evidence varies in how it is generated, documented and perceived to apply to decision contexts, yet approaches for systematically describing these variations remain underdeveloped.

Against this backdrop, this article describes the development of a spectrum approach that aims to map the diverse types of evidence used in health policy decision-making in relation to (i) the nature of the evidence – specifically, where it sits on a continuum from tacit to scientific and (ii) its geographic scope – whether it represents global or local sources of knowledge. The first dimension captures different ways in which evidence is generated, while the second dimension captures the perceived relevance and contextual applicability of evidence generated elsewhere. By positioning evidence along these two continua, the approach offers a way to more systematically describe and compare evidence used in decision-making and to make more transparent how different evidence sources can relate to the decision context. Previous frameworks have provided ways of thinking about other dimensions of evidence use, such as the purpose of use (e.g. instrumental, conceptual or political) [9], or the nature of decision-maker questions (e.g. effectiveness, acceptability, health equity, human rights and social justice) [36]. Our approach seeks to add to and complement these other frameworks.

## Methods

**Fig. 1:**
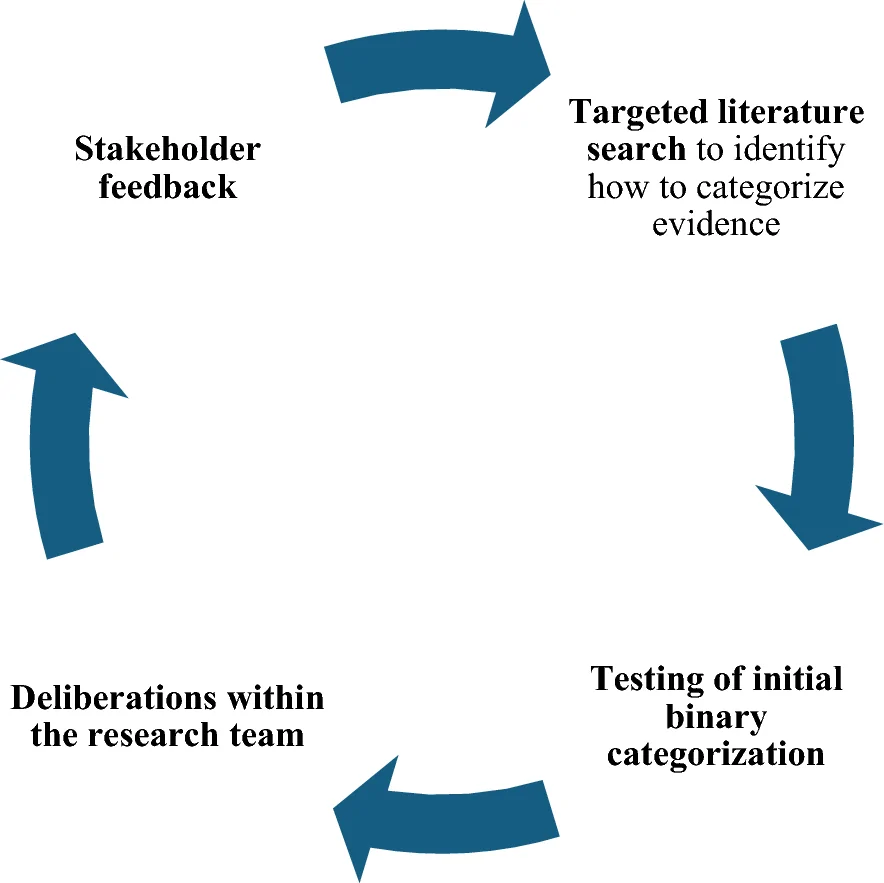
Stages involved in developing the spectrum approach

We developed the spectrum approach in four stages (Fig. 1). The process was iterative, with insights from later stages prompting us to revisit and refine earlier steps.

### Stage 1: Targeted literature search

In this stage, we identified key papers within the field of health policy and systems research that defined and/or categorized the concept of “evidence”. The search was guided by expert recommendations and supplemented through screening reference lists from the suggested papers. From the identified literature, we extracted descriptions of how evidence was defined and/or categorized (Table 1).

**Table 1:** Summary of selected papers and key findings on how evidence is defined and categorized for decision-making

Articles guiding evidence use	Type of article	Aim	Evidence definitions, categorization and/or operationalization
1. Lomas et al. [28]	Literature review	To examine how the concept of evidence is treated by those who produce scientific evidence, those who formulate guidance and those who make decisions	Defines evidence as “facts intended to support a conclusion”. Distinguishes evidence by methodological rigour (scientific) versus practical/colloquial sources; separates scientific evidence on effectiveness from scientific evidence on contextual factors (implementation, economics and ethics). Notes decision-makers prioritize practical utility, whereas researchers prioritize methodological rigour, highlighting tensions between context-sensitive and hierarchy-oriented approaches to evidence use
2. Oxman et al. [2]	Guidance document	To define evidence, evidence-informed policy-making and the role of research evidence in informing health policy decisions	Adopts Lomas et al.’s definition of evidence, and frames evidence-informed policy-making as use of the “best available research evidence”. Categorizes evidence by method/quality (scientific versus haphazard), and by geographical origin (global versus local), while recognizing that policy-making also involves considerations beyond research evidence alone
3. Lewin et al. [37]	Guidance document	To describe how to find and use evidence about local conditions	Focuses on evidence about local conditions; distinguishes global versus local evidence and provides methods to locate and use local data in policy decisions
4. Kothari et al. [18]	Empirical	To explore the use of tacit knowledge in programme planning in public health	Contrasts explicit/scientific knowledge with implicit/tacit knowledge in public health planning; operationalizes evidence as either codified research or practitioner experience
5. Swan et al. [32]	Empirical	To understand practices and conditions that enable evidence use in commissioning work	Defines evidence as “information with evidentiary value” and classifies it into universal (e.g. scientific, guidelines and routine data) local, expertise-based and trans-local forms of evidence
6. WHO [5]	Guidance document	To provide guidance to WHO staff, Member States and in partner organizations who need to create, commission, fund, broker or apply evidence	Defines evidence as factual knowledge from observation/experiment; emphasizes use of best available research plus consideration of contextual factors (equity, feasibility and acceptability). Classifies evidence by method (scientific versus tacit) and geographic origin (global versus local)
7. Glenton et al. [38]	Guidance document	To describe the process used to broaden the range of evidence used to develop WHO’s recommendations regarding the use of task-shifting strategies for key maternal and newborn interventions	Does not provide a definition of evidence but documents how the WHO broadened the evidence base used in guidance development by incorporating qualitative syntheses on acceptability and feasibility alongside effectiveness evidence
8. Lewin et al.. [23]	Guidance document	To describe the development and content of the Assessing Unconventional Evidence (ACE) tool	Adopts Lomas et al.’s definition of evidence; distinguishes scientific (primary/secondary research) from unconventional or real-world evidence (stakeholder documents and experiential sources) and provides an assessment tool for such materials, thereby broadening how evidence for decision-making may be identified and assessed
9. Parkhurst [10]	Guidance document	To demonstrate the limitations of using a single evidence hierarchy to guide health policy decisions and to propose an alternative framework that redefines so-called good evidence as contextually appropriate evidence for achieving health sector goals	Argues against single evidence hierarchies; frames so-called good evidence as contextually appropriate. Does not adopt a rigid definition but critiques unitary hierarchies and promotes appropriateness over method-based evidence ranking
10. Mansilla et al. [36]	Empirical	To create a taxonomy of demand-driven questions for use by evidence producers, intermediaries (i.e. people working in between researchers and decision-makers) and decision-makers	Draws on an inclusive definition of evidence (encompasses all relevant decision-making data such as modelling, evaluations, qualitative insights, operational research, guidelines and technology assessments) and provides a taxonomy of 41 demand-driven policy questions where evidence could provide decision-makers with insights. Rather than classifying evidence itself, this paper offers an alternative way of organizing evidence needs around the types of questions decision-makers ask

Most papers classified evidence along two recurring binary dimensions: (i) its nature, distinguishing scientific from tacit or experiential forms of knowledge [18, 23, 28], and/or (ii) its geographic scope, contrasting globally generated with locally grounded evidence [2, 32, 37]. Some papers also highlighted hierarchy-based ways of classifying evidence according to methodological rigour, and critiqued these by calling for more context-sensitive and inclusive conceptualizations of evidence for health policy [2, 10, 28]. Despite these calls, we found that the reviewed literature did not offer a unifying framework with a structured way of moving beyond these binaries in health policy and systems decision-making. We therefore used the tacit–scientific and global–local distinctions as the initial binary categorization of evidence in the next stage of framework development.

### Stage 2: Testing of initial binary categorization

In this stage, we tested the adequacy of the two recurring binary distinctions (tacit–scientific and global–local) identified during stage 1 using empirical material from two sources, described further in Box 1:

(i) Findings from a Cochrane review on civil society evidence use in health policy decision-making [39] and
(ii) Qualitative data from an empirical study examining evidence use in vaccine policy-making in Kenya [40].

**Table:** Box 1: Overview of the two empirical sources used in stage 2

Empirical source	Brief description and methods	How the source was used in this paper
Cochrane review on civil society evidence use in health policy decision-making [39]	A systematic review exploring how evidence from civil society actors is brought into national and subnational health policy processes. Eligible studies included in the review report empirical data on how civil society actors contributed, communicated or mobilized evidence in health policy processes.	We selected a sample of the studies included in the systematic review, and used the types of evidence described in these studies to test a binary tacit–scientific distinction. This showed that this distinction provided an incomplete portrayal of the evidence. Many of the evidence types included in these studies cut across the binary categorization and met different levels of transparency and reproducibility, ranging from structured collection, coding and analysis of community feedback to more informal, mediated or ad hoc use of community perspectives. Illustrative examples are presented in Box 2.
Kenyan case study on evidence use in vaccine policy-making in Kenya [40]	A qualitative case study exploring evidence use in national vaccine policy-making in Kenya, focusing on the introduction and roll-out of human papilloma virus (HPV) and malaria vaccines. Data included 34 in-depth interviews, 417 hours of observation of vaccine-related policy and planning meetings, and a review of relevant policy and planning documents.	We applied the binary tacit–scientific categorization to the types of evidence used in this primary study. As above, this assessment showed that this evidence could not be adequately categorized as either tacit or scientific but needed to be considered along a continuum from tacit to scientific and from local to global. Examples from this primary study were further used to illustrate how the spectrum approach can be applied to evidence used in HPV and malaria vaccine policy-making in Kenya (Fig. 4 and Table 2).

Using the two empirical sources described in Box 1, we examined how examples of evidence varied in their characteristics and use, and assessed how well the binary categories captured the diversity of evidence used in practice.

Both sources revealed substantial challenges in distinguishing between different types of evidence based on these binary categories. For example, the Cochrane review identified wide variation in the systematization, transparency and reproducibility of evidence representing civil society’s opinions, values and practices. Some forms – such as structured community feedback systems – showed high levels of methodological rigour, aligning closely with scientifically generated qualitative evidence. Others relied on informal or opaque processes, with minimal documentation or replicability. Box 2 illustrates these variations using two cases from the Cochrane review described in Box 1.

**Table:** Box 2: Illustration of the variations in how evidence on civil society views, opinions and practices is produced from the Cochrane review findings

Case 1: Structured use of community feedback in the Ebola response, Democratic Republic of Congo [41] During the Ebola response in the Democratic Republic of Congo, the International Federation of Red Cross and Red Crescent Societies (IFRC) established a structured community feedback system to capture, analyse and act on public concerns. Red Cross volunteers collected unstructured feedback and comments from communities during routine interactions, classifying it into six groups: (1) questions; (2) statements (rumour, belief, observation); (3) suggestions/requests; (4) sensitive or violence related; (5) appreciation; and (6) other (e.g. refused dialogue). These were reviewed by local teams, thematically coded by IFRC staff and volunteers and quality checked by the United States Centers for Disease Control and Prevention (CDC). Through iterative coding and joint validation meetings, the process evolved into a system that produced weekly briefs, trend analyses and dashboards used to inform strategic and operational decisions. Case 2: Informal and ad hoc incorporation of service user views in housing policies for people with mental health issues, South Australia (2000–2005) [42] Battams and Johnson’s [42] study of the influence of service users on housing policies for people with mental health issues in South Australia between 2000 and 2005 reveals a markedly less systematic and transparent use of community perspectives [37]. Despite formal commitments to consumer participation, user and carer influence on housing policy for people with psychiatric disabilities remained limited and largely mediated through nongovernmental organizations or professional advocates. Policy-makers appeared to rely more heavily on informal channels, including views aired through the media – particularly a prominent radio presenter – as proxies for community sentiment. This approach to seeking and using evidence lacked transparent methods for data collection, validation or analysis, and offered little reproducibility in how community perspectives shaped policy decisions.	

Applying the categorization to the data from Kenya similarly demonstrated the inadequacy of binary categories. For example, WHO guidance documents and local programme data, commonly used to inform new vaccine programme design and roll-out activities [40], presented particular challenges in categorizing evidence as either scientific or tacit. Some WHO guidance documents, including the WHO position papers, contained explicit documentation of the evidence that informed it (e.g. research studies or systematic reviews) and how this evidence was used to inform conclusions, including the role of expert judgements. In comparison, other WHO guidance documents, such as WHO’s training materials for health workers, lacked comprehensive information about their evidence base and decision-making processes. Some of the local programmatic data (e.g. cold chain capacity assessments) also proved problematic to classify as either scientific or tacit evidence as these were more structured than experiential knowledge yet lacked the rigour of formal research studies. Additionally, the global–local distinction proved oversimplified, as the Kenyan decision-makers considered evidence from neighbouring African countries or countries with similar economic status (i.e. low- and middle-income countries) more relevant than similar evidence from other regions [40]. Overall, stage 2 showed that binary tacit–scientific and global–local distinctions were insufficient to capture the diversity of evidence used in practice and to make transparent how different evidence sources varied in their generation and contextual applicability.

### Stage 3: Iterative researcher deliberations

**Fig. 2:**
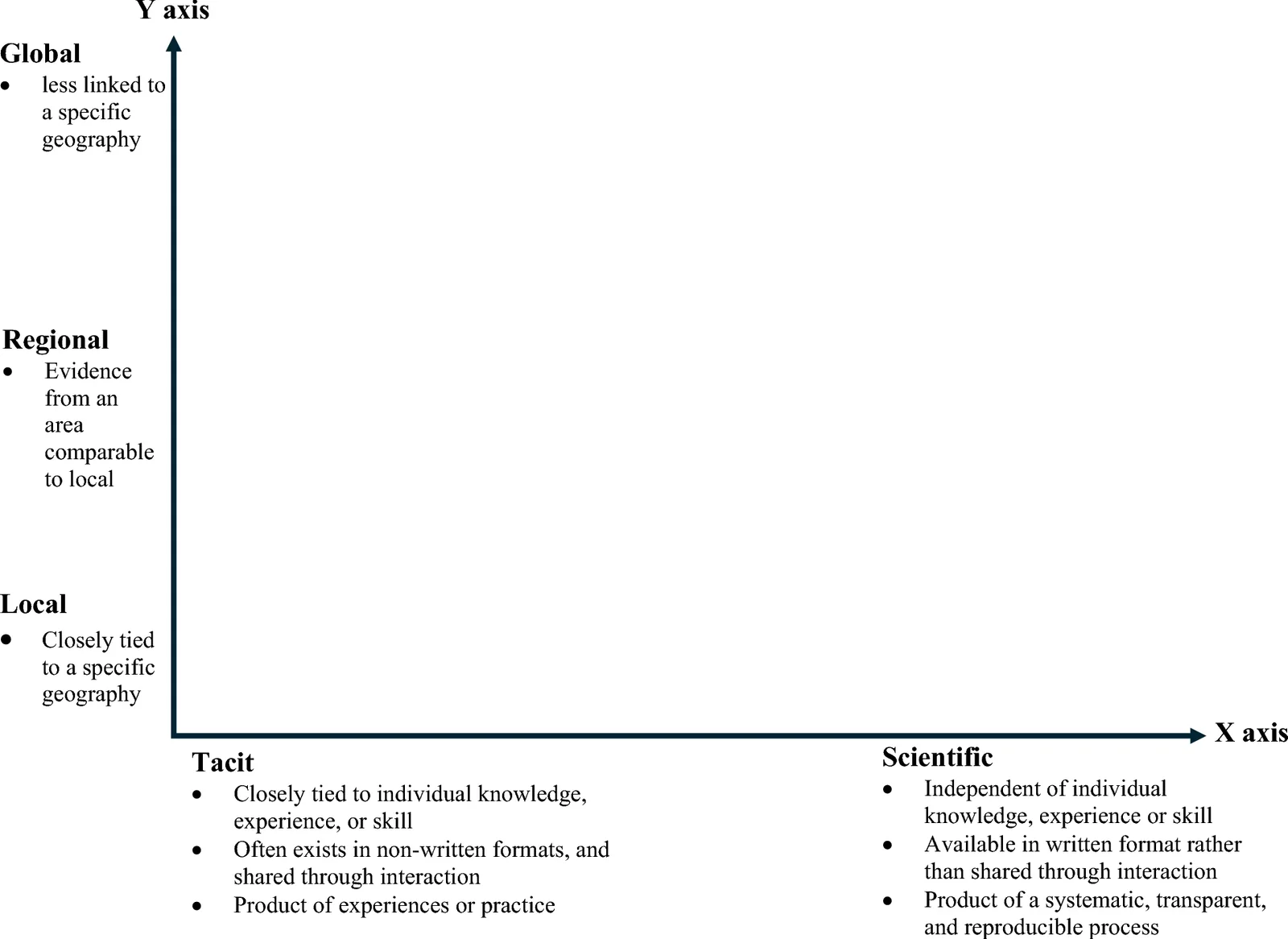
Spectrum approach to evidence categorization with markers for mapping against the *x*- and *y*-axis

The research team engaged in iterative deliberations to address the categorization challenges identified during stage 2, which highlighted two key insights. First, we identified the need for a framework that simultaneously positions evidence along the tacit–scientific (*x*) and global–local (*y*) axes, allowing different sources to be located in relation to both dimensions (Fig. 2). Second, the team recognized the need to position evidence at different points along each axis (Fig. 2). This allowed us to move beyond the binary categorizations identified in stage 1 while retaining the two dimensions that most consistently appeared in the reviewed literature. The *x*-axis ranges from tacit to scientific evidence. As evidence moves towards the scientific end of the continuum, it is increasingly likely to be different in terms of these markers:

- Less reliant on a single person’s knowledge, experiences or skills [18].
- Available in written format rather than shared through interaction [18].
- The product of a systematic, transparent and reproducible process (e.g. as indicated by a protocol, a data analysis plan, systematic approaches to data collection and/or analysis described transparently, descriptions of participants, among others) [2, 5].

On the *y*-axis, ranging from global to local, evidence positioned towards the global end is characterized by being less linked to a specific geography, while evidence towards the local end is closely tied to a specific geography (Fig. 2) [2, 5, 37]. Between the global and local ends of the spectrum is regional or comparable evidence, i.e. evidence that is from an area comparable to the local (or decision-making) context. This could be evidence from the same geographical region or from a healthcare system in another region that is otherwise comparable in its organization, governance and/or financing. Together, these markers provided a systematic basis for mapping diverse forms of evidence on each axis.

### Stage 4: Stakeholder feedback

As part of a larger research project on inclusive and accountable health systems decisions [43], we had a number of meetings with stakeholders to share research results. These stakeholder engagements included the annual meeting of the project advisory body (comprising policy-makers, academic experts, members of national advisory committees, and staff from regional and multilateral organizations); sessions at the Global Evidence Summit 2024 and the Global Symposium on Health Systems Research 2024; and discussions with biomedical and social science experts at the Norwegian Institute of Public Health (Departments of Infection Control and Vaccines, and Global Health). We used these forums to introduce the spectrum approach, show its application to the different types of evidence collected in the wider study [40] and obtain feedback on how we had mapped these evidence types as well as on the conceptual clarity and practical applicability of the approach. These interactions provided an opportunity to elicit reflections and feedback from diverse audiences. In total, approximately 40 participants representing a range of disciplines and geographic regions engaged with the framework and provided feedback. Some stakeholders noted that the approach helpfully broadens understanding of the different types of evidence used in health policy-making, including contributions from local actors whose inputs are often overlooked. Others, however, highlighted that it does not explicitly distinguish between more and less trustworthy forms of evidence. As the aim of the spectrum is to map the range and positioning of evidence rather than the methodological rigour, this feedback was useful in clarifying the scope of the approach, and we used such reflections to further refine its presentation.

## Results

**Fig. 3:**
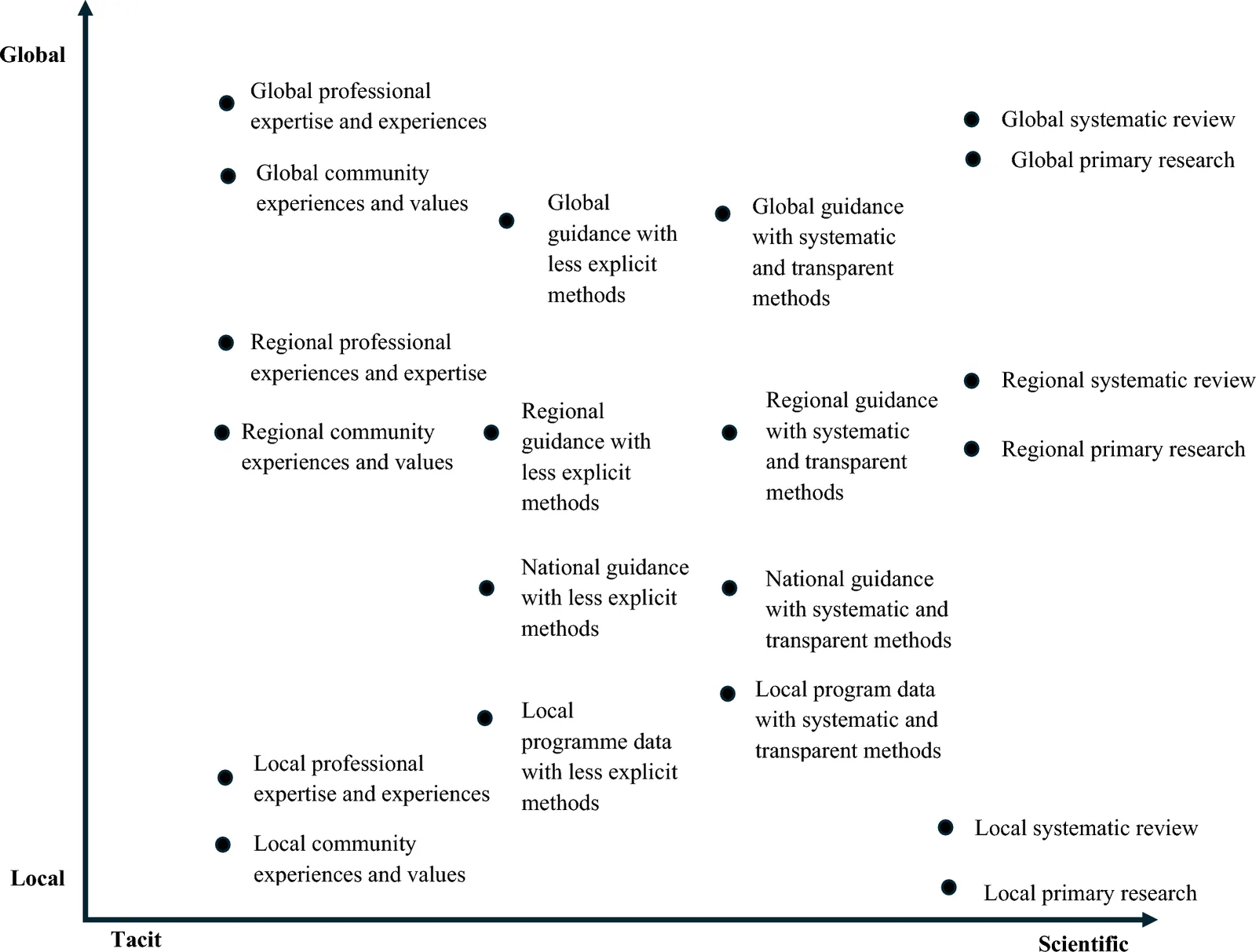
Mapping of different evidence types in the framework

Figure 3 shows how different types of evidence identified from both the literature and the Kenyan empirical data (Box 1) were mapped in a framework that includes the tacit–scientific and global–local axes.

Mapping along the *x*-axis was guided by the tacit–scientific markers (Fig. 2). Evidence positioned towards the scientific end (right) was characterized by systematic, transparent and reproducible methods of knowledge generation that were largely independent of individual experience. Conversely, evidence at the tacit end (left) drew heavily on experiential, context-specific knowledge, often transmitted through personal interactions. Evidence types exhibiting intermediate levels of systematization, transparency or independence from individual judgment were positioned near the midpoint of the axis. For example, some forms of global guidance, although generally explicit about their evidence base and the systematic processes through which recommendations are developed, also rely on expert interpretation and consensus, introducing an element of subjectivity. These are therefore positioned mid-right (towards the scientific end) rather than at the extreme right. In contrast, global guidance in which both the underlying evidence and development processes are described less transparently are positioned mid-left (towards the tacit end).

Mapping along the *y*-axis was informed by the local–global markers (Fig. 2). Evidence positioned towards the global end (top) was characterized by not being tied to specific geographical settings, such as global systematic reviews or global guidance. Conversely, evidence towards the local end (bottom) was directly rooted in the geographical setting of those making the decision, such as local research or local programme data. Between these two ends lies regional or comparable evidence, derived from settings with comparable geographic or health systems conditions, such as regional research or regional guidance.

**Fig. 4:**
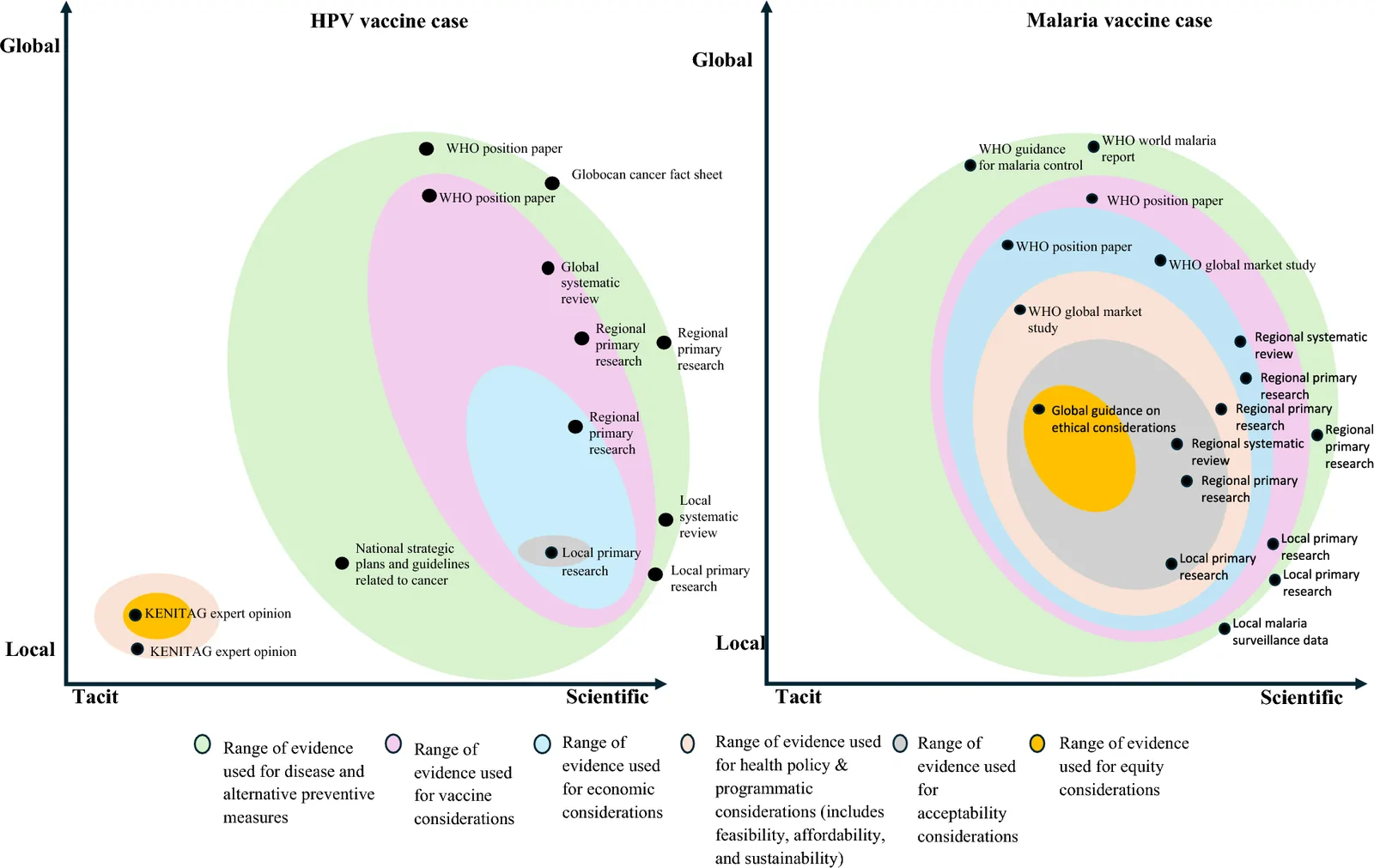
Range of evidence used by KENITAG’s working groups for HPV and malaria vaccine programme discussions

In Fig. 4, we further illustrate the application of the spectrum approach to empirical findings from the earlier mentioned Kenyan case study on evidence use in vaccine policy-making (Box 1) [40]. The mapping focused on evidence use by the Kenya National Immunization Technical Advisory Group (KENITAG) in its recommendations to introduce both human papilloma virus (HPV) and malaria vaccines. KENITAG is an independent expert committee that reviews evidence to advise the Kenyan Ministry of Health on vaccine policies and introduction decisions within the national immunization program [40]. Malaria vaccine discussions drew on a broader range of global, regional and local scientific evidence across the different considerations, such as economic impacts, acceptability and equity (coloured circles in Fig. 4), whereas the HPV case sometimes relied solely on local expert opinions or single studies, indicating narrower evidence breadth (also shown by coloured circles in the adjacent graph of Fig. 4). For example, WHO position papers were mapped as global and mid-right because they not only drew on systematic review processes but also involved expert interpretation and consensus. Kenyan cancer policy documents were mapped as local and mid-left because they reflected national programme data and institutional judgement, but with less transparent methods. Local malaria surveillance data were mapped as local and scientific, while KENITAG expert opinion on equity and feasibility in the HPV case was mapped as local and tacit. These examples show that vaccine decisions may require evidence across both dimensions of the spectrum: from tacit to scientific evidence, and from local to global evidence. Overall, applying the spectrum approach to the Kenyan case showed that it can be used not only to show the breadth of evidence drawn on across the entire decision-making process but also to identify and reflect on how different decision considerations were informed by evidence that occupy different positions along the tacit–scientific and global–local dimensions. Supplementary Files 1 and 2 provide further details for the HPV and malaria vaccine cases, respectively, regarding the evidence shown in Fig. 4.

Drawing from Fig. 4, Table 2 below provides illustrative examples of how users can apply the spectrum approach to make judgements about where different types of evidence should be positioned. Each example is assessed against the previously described markers to determine its position along both the tacit–scientific and global–local axes.

**Table 2:** Explanation of evidence positioning in the spectrum approach (based on examples from Fig. 4)

Evidence type	Was a systematic process used to generate or analyse the evidence?	Was the evidence independent of individual knowledge?	Was the evidence documented in a written format?	What was the geographical or comparable context?	*X*-axis position (scientific ↔ tacit)	*Y*-axis position (global ↔ local)	Explanation for position
WHO position paper (HPV [44] and malaria [45])	Yes	Partly – expert judgements influence final recommendations	Yes	Global (applied across different contexts)	Middle-right (towards scientific end)	Global (top)	Based on systematic reviews and formal appraisal processes, but final recommendations rely on expert interpretation and consensus
National Cervical Cancer Strategic Plan [46] and National Cancer Guideline [47]	No – largely policy- and context-driven	Partly – informed by expert and institutional priorities as well as programme and research data	Yes	Kenya	Middle-left (towards tacit end)	Local	Draw on existing data and policy priorities but not developed through transparent or standardized methods
Local malaria surveillance data [48]	Yes	Yes	Yes	Kenya	Scientific (right)	Local	Systematically generated from routine surveillance and analysis within Kenya’s health system
KENITAG expert opinion on equity and feasibility (HPV)	No	No – grounded in experiential and contextual judgement	No	Kenya	Tacit (left)	Local	Based on professional experience and deliberation rather than formalized, reproducible methods; contextually rich but informal

## Discussion

We have described the development of a nuanced spectrum approach to mapping evidence, designed to move from viewing evidence as binary along global–local and tacit–scientific dimensions to viewing it as positioned along a continuum within each of these dimensions. Some frameworks developed outside the field of health policy and systems research have also taken more nuanced approaches to assessing research quality beyond binary or hierarchy-based models. Specifically, the International Development Research Centre’s Research Quality Plus (RQ+) framework assesses research quality through four quality dimensions: scientific rigour, research legitimacy, research importance and positioning for use [49]. Within this framework, the research legitimacy dimension prompts evaluators to consider how well the research engages with and respects local knowledge systems and contexts. Attention to inclusiveness and user involvement is also reflected across other dimensions, particularly research importance and positioning for use. However, RQ+ was developed primarily as an evaluative framework for assessing the quality of development research projects and their positioning for use [49].

In comparison, the spectrum approach is intended as a conceptual and practical tool for mapping the types of evidence drawn on in health policy and systems decision-making and locating them simultaneously along tacit–scientific and global–local dimensions. In this way, the spectrum approach contributes conceptually to ongoing debates about how evidence is understood in health policy and systems research [2, 5–7, 10, 11, 19, 50, 51]. More concretely, the spectrum approach recognizes that both global and local sources of evidence can be more or less systematic, transparent and reproducible, and may also vary in the extent to which they are shaped by individual subjective influences, such as practitioner-based insights or expert opinion. By moving beyond binary categorizations, the approach does not privilege some types of evidence over others through an uncritical use of traditional evidence hierarchies or pyramids [10, 19].

Beyond its conceptual contribution, the spectrum approach offers practical value for both researchers and decision-makers by helping them consider different types of evidence that can be complementary during health policy-making [2, 5, 37]. For researchers, it presents a structured lens for systematically mapping the breadth of evidence informing policy decisions, thereby identifying patterns of dominance or neglect of some types of evidence [6, 10, 11, 35, 50]. Moreover, it offers researchers a tool for critically reflecting on the range of evidence included in literature reviews. For example, when conducting global systematic reviews to inform assessments of a health programme’s acceptability or feasibility, it may be relevant to include insights from government programme reports or field reports produced by civil society actors that are not usually published in peer-reviewed journals. For decision-makers, it may function as a visual reflective aid to assess what forms of global and local evidence are available and where critical gaps exist. Global evidence can offer insights on the potential impacts of new interventions or policies, while different types of local evidence can simultaneously help decision-makers translate these insights into context appropriate decisions and actions [2, 5, 37]. Tacit evidence can support decision-makers in assessing the applicability of scientific findings and adding context-specific insights, often rooted in people’s individual experiences and knowledge [5], and may therefore be especially useful for informing programme implementation and adaptation within specific contexts.

At the same time, the spectrum approach helps decision-makers recognize that local evidence is not synonymous with “tacit” evidence: It can take both scientific forms (such as operational research) and tacit forms (such as community stories). Similarly, global evidence may be either scientific (e.g. multicountry randomized trials) or a combination of more and less systematic, transparent and reproducible sources of knowledge (e.g. global advocacy reports). Moreover, the spectrum approach highlights the value of integrating global and local evidence, as corroborating locally generated evidence with experiences from other settings can strengthen the basis for decisions – particularly where local evidence carries methodological uncertainty – and reduce the risk of overlooking credible evidence on relevant approaches and implementation experiences that may inform local decision-making [37].

Finally, we recognize three key limitations of the tool. First, while the spectrum approach offers a more inclusive basis for mapping evidence, it does not prescribe how different types of evidence should be weighted or combined in decision-making. Such weighting depends on the policy question being considered and normative and contextual considerations that may vary across policy domains [52]. It also depends on the methodological strengths and limitations of the evidence source, which needs to be assessed using other available tools [23, 53, 54]. Second, while we developed criteria to support the positioning of evidence along the two axes, applying these criteria still requires judgement and may therefore be open to subjectivity and bias. We therefore anticipate variation in how different users may place the same evidence on the spectrum. Third, additional dimensions of evidence emphasized in broader literature, such as the participatory nature of evidence generation [52], the purpose of use (e.g. instrumental, conceptual or political) [9] or the nature of decision-maker questions (e.g. effectiveness, acceptability, health equity, human rights and social justice) [36], are not captured within this version of the spectrum approach. Future work could explore whether an expansion of the approach to include any of these dimensions is possible and worthwhile and empirically test its application in real-world decision-making settings. This might include participatory studies to examine how diverse stakeholders interpret and use the spectrum approach, and methodological work exploring its complementarity with existing tools for assessing methodological strengths and limitations [54, 55]. Such refinements would ensure the approach remains responsive to the complex realities of evidence use in health policy-making and continues to evolve as a practical and conceptual contribution to the study of evidence-informed decision-making.

## Conclusions

This article has introduced the spectrum approach to move beyond the binary or hierarchical categorizations of evidence that often dominate health policy and systems research. By positioning different types and sources of evidence along intersecting axes that capture their nature and origin, the spectrum approach offers a dynamic way to visualize the diverse forms of evidence that can be valuable for health systems decision-making. It contributes theoretically by promoting a more nuanced understanding of evidence, and practically as a heuristic for researchers and decision-makers to consider and map multiple forms of knowledge – contributing towards bridging the divide between global and local knowledge systems and supporting more grounded, inclusive and transparent evidence use in policy and practice.

## Supplementary Information

Supplementary material 1.

Supplementary material 2.

## Data Availability

No new datasets were generated or analysed for this study. The conceptual approach was informed by published literature and findings from a previously reported study. All materials necessary to understand and apply the spectrum approach are included within the manuscript and its supplementary files. Additional information is available from the corresponding author upon request.

## Abbreviations

WHO: World Health Organization
ACE: Assessing Unconventional Evidence
IFRC: International Federation of Red Cross and Red Crescent Societies
CDC: Centers for Disease Control and Prevention
KENITAG: Kenya National Immunization Technical Advisory Group
HPV: Human papilloma virus
