# Supplementary material for: Mapping evidence for health policy and systems decision-making: a spectrum approach bridging tacit and scientific knowledge across local and global contexts
Source: Health Res Policy Syst. 2026 Jun 30;24:79. doi: 10.1186/s12961-026-01502-4 (PMC13591811; doi:10.1186/s12961-026-01502-4)
Supplement: Supplementary file 1 [file 12961_2026_1502_MOESM1_ESM.docx]

Table 1: Detailed description of the evidence used to inform KENITAG's working group for HPV vaccine programme discussions

| **Consideration** | **Document citation** | **Aim** | **Type (as referred to in Figure 4)** |
| --- | --- | --- | --- |
| **Disease and alternative preventive measures** | Gichangi, Bwayo, Estambale, De Vuyst, Ojwang, Rogo, Abwao, Temmerman ^1^. | To determine the association between invasive cervical cancer (ICC) and HIV infection in Kenyan women. | Local primary research |
|  | KNBS, NASCOP ^2^ | To evaluate HIV care, treatment, and prevention programmes and to inform new strategies to respond to the HIV epidemic in Kenya. | Local primary research |
|  | De Vuyst, Mugo, Chung, McKenzie, Nyongesa-Malava, Tenet, Njoroge, Sakr, Meijer, Snijders, Rana, Franceschi ^3^ | To assess the prevalence and determinants of HPV infection and cervical lesions in HIV-positive women in Kenya | Local primary research |
|  | Ermel, Qadadri, Tong, Orang'o, Macharia, Ramogola-Masire, Zetola, Brown ^4^. | To compare HPV typing data from a previously published study of ICC cases in the United States and Botswana to additional cases identified in a second sub-Saharan country, Kenya, and to determine the HPV type distribution in invasive cervical cancers from HIV-infected women living in these countries. | Local primary research |
|  | Menon, Wusiman, Boily, Kariisa, Mabeya, Luchters, Forland, Rossi, Callens, Vanden Broeck ^5^ | To report on the prevalence of pHR/HR HPV types and multiple pHR/HR HPV genotypes in Kenya among HIV positive women with normal, abnormal cytology and invasive cervical cancer. | Local systematic review |
|  | Coleman, Cespedes, Cu-Uvin, Kosgei, Maloba, Anderson, Wilkin, Jaquet, Bohlius, Anastos, Wools-Kaloustian ^6^ | To assess cervical cancer prevention capacity in select HIV clinics located in resource-constrained countries. | Regional primary research |
|  | WHO ^7^ | To replace the first 2009 HPV vaccine WHO position paper based on emerging findings. The main change from the previous WHO recommendations on the use of the HPV vaccine was that recent evidence supported shift from a three-dose administration schedule to a two-dose schedule. | WHO position paper |
|  | Globocan ^8^ | To provide a summary of cervical cancer incidence, mortality and prevalence worldwide | Globocan cancer fact sheet |
|  | MoH ^9^ | To provide the strategic framework and priority actions for cervical cancer prevention and control | National cervical cancer strategic plan |
|  | MoH ^10^ | To provide the standards for comprehensive reproductive tract cancer prevention and management | National guidelines related to cancer |
| **Vaccine** | Mugo, Ansah, Marino, Saah, Garner ^11^ | To assess the safety and immunogenicity of a quadrivalent human papillomavirus (qHPV) vaccine in sub-Saharan African women | Regional primary research |
|  | Malagón, Drolet, Boily, Franco, Jit, Brisson, Brisson ^12^ | Cross-protective efficacy of two human papillomavirus vaccines: a systematic review and meta-analysis | Global systematic review |
|  | WHO ^7^ | To replace the first 2009 HPV vaccine WHO position paper based on emerging findings. The main change from the previous WHO recommendations on the use of the HPV vaccine was that recent evidence supported shift from a three-dose administration schedule to a two-dose schedule. | WHO position paper |
| **Economic considerations** | Campos, Kim, Castle, Ortendahl, O'Shea, Diaz, Goldie ^13^ | To assess the cost-effectiveness of prevention strategies, estimate the financial costs of preadolescent HPV vaccination, and explore alternative assumptions about vaccine efficacy,  coverage, and duration of protection, as well as the impact of screening coverage, test performance, and loss to follow-up. | Regional primary research |
| **Health policy and programmatic considerations (includes feasibility)** | Not applicable | Not applicable | Expert opinion (based on KENITAG deliberations) |
| **Acceptability** | HPV pilot evaluation report (unpublished; cited in KENITAG recommendation report) | To inform discussions on coverage, costs, and acceptability based on pilot implementation experience in Kenya | Local primary research |
| **Equity** | Not applicable | Not applicable | Expert opinion (based on KENITAG deliberations) |

# **References**

**1.**	Gichangi PB, Bwayo J, Estambale B, et al. Impact of HIV infection on invasive cervical cancer in Kenyan women. *Aids.* 2003;17(13):1963-1968. doi:10.1097/00002030-200309050-00015

**2.**	KNBS, NASCOP. Kenya Aids Indicator Survey 2012. In: (NASCOP) KNBoSKaNAaSCP, ed2012.

**3.**	De Vuyst H, Mugo NR, Chung MH, et al. Prevalence and determinants of human papillomavirus infection and cervical lesions in HIV-positive women in Kenya. *Br J Cancer.* 2012;107(9):1624-1630. doi:10.1038/bjc.2012.441

**4.**	Ermel A, Qadadri B, Tong Y, et al. Invasive cervical cancers in the United States, Botswana and Kenya: HPV type distribution and health policy implications. *Infect Agent Cancer.* 2016;11:56. doi:10.1186/s13027-016-0102-9

**5.**	Menon S, Wusiman A, Boily MC, et al. Epidemiology of HPV Genotypes among HIV Positive Women in Kenya: A Systematic Review and Meta-Analysis. *PLoS One.* 2016;11(10):e0163965. doi:10.1371/journal.pone.0163965

**6.**	Coleman JS, Cespedes MS, Cu-Uvin S, et al. An Insight Into Cervical Cancer Screening and Treatment Capacity in Sub Saharan Africa. *J Low Genit Tract Dis.* 2016;20(1):31-37. doi:10.1097/lgt.0000000000000165

**7.**	WHO. Human papillomavirus vaccines : WHO position paper, October 2014 = Vaccins contre le papillomavirus humain : note de synthèse de l’OMS, octobre 2014. *Weekly Epidemiological Record = Relevé épidémiologique hebdomadaire.* 2014;89(43):465-491.

**8.**	Globocan. *Globocan cancer fact sheets:cervical cancer. Cervical cancer incidence, mortality and prevalence worldwide in 2008 summary.*2008,.

**9.**	MoH. National Cervical Cancer Prevention Strategic Plan (NCCPSP)2012.

**10.**	MoH. National Guidelines for Prevention and Management of Cervical, Breast and Prostate Cancers2012.

**11.**	Mugo N, Ansah NA, Marino D, Saah A, Garner EI. Evaluation of safety and immunogenicity of a quadrivalent human papillomavirus vaccine in healthy females between 9 and 26 years of age in Sub-Saharan Africa. *Hum Vaccin Immunother.* 2015;11(6):1323-1330. doi:10.1080/21645515.2015.1008877

**12.**	Malagón T, Drolet M, Boily MC, et al. Cross-protective efficacy of two human papillomavirus vaccines: a systematic review and meta-analysis. *Lancet Infect Dis.* 2012;12(10):781-789. doi:10.1016/s1473-3099(12)70187-1

**13.**	Campos NG, Kim JJ, Castle PE, et al. Health and economic impact of HPV 16/18 vaccination and cervical cancer screening in Eastern Africa. *Int J Cancer.* 2012;130(11):2672-2684. doi:10.1002/ijc.26269
