# Supplementary material for: Mapping evidence for health policy and systems decision-making: a spectrum approach bridging tacit and scientific knowledge across local and global contexts
Source: Health Res Policy Syst. 2026 Jun 30;24:79. doi: 10.1186/s12961-026-01502-4 (PMC13591811; doi:10.1186/s12961-026-01502-4)
Supplement: Supplementary file 2 [file 12961_2026_1502_MOESM2_ESM.docx]

Table 1: Detailed description of the evidence used to inform KENITAG's working group for Malaria vaccine programme discussions

| **Consideration** | **Document citation** | **Aim** | **Type (as referred to in Figure 4)** |
| --- | --- | --- | --- |
| **Disease and alternative preventive measures** | MOH ^1^ | To provide data for monitoring core malaria control interventions in Kenya, including malaria prevalence, anaemia, and household ownership, access to, and use of insecticide-treated nets | Local malaria surveillance data |
|  | Macharia, Giorgi, Noor, Waqo, Kiptui, Okiro and Snow ^2^ | To conduct a spatio‑temporal analysis of Plasmodium falciparum prevalence in Kenya. | Local primary research |
|  | Alegana, Suiyanka, Macharia, Ikahu-Muchangi and Snow ^3^ | To depict sub-national heterogeneities in test positivity rate (TPR) for malaria among patients attending health facilities in 8 counties in Western Kenya. | Local primary research |
|  | Alegana, Macharia, Muchiri, Mumo, Oyugi, Kamau, Chacky, Thawer, Molteni, Rutazanna, Maiteki-Sebuguzi, Gonahasa, Noor and Snow ^4^ | To analyse plasmodium falciparum parasite prevalence in East Africa. | Regional primary research |
|  | Kamau, Paton, Akech, Mpimbaza, Khazenzi, Ogero, Mumo, Alegana, Agweyu, Mturi, Mohammed, Bigogo, Audi, Kapisi, Sserwanga, Namuganga, Kariuki, Otieno, Nyawanda, Olotu, Salim, Athuman, Abdulla, Mohamed, Mtove, Reyburn, Gupta, Lourenço, Bejon and Snow ^5^ | To explore the relationships between age and severity of malaria hospitalisation versus malaria transmission intensity in East Africa. | Regional primary research |
|  | WHO ^6^ | To describe: the global distribution of malaria cases and deaths; adoption of WHO recommended control strategies in endemic countries; sources of funding for malaria control; and emerging evidence on the impact of strategies adopted. | WHO world malaria report |
|  | WHO ^7^ | To outline WHO recommendations for malaria control including the evidence synthesis | WHO guidance for malaria control |
| **Vaccine** | RTSS Clinical Trials Partnership ^8^ | To assess the efficacy and safety of RTS,S/AS01 malaria vaccine with or without a booster dose in infants and children in Africa. | Regional primary research |
|  | WHO ^9^ | To assess the malaria vaccine safety, impact (includes cost-effectiveness) and feasibility in the context of routine immunisation programmes. | Regional primary research |
|  | WHO ^10^ | To outline WHO’s recommendations on the use of malaria vaccine. | WHO position paper |
| **Economic considerations** | Penny, Verity, Bever, Sauboin, Galactionova, Flasche, White, Wenger, Van de Velde, Pemberton-Ross, Griffin, Smith, Eckhoff, Muhib, Jit and Ghani ^11^ | To assess the public health impact and cost-effectiveness of routine use of the RTS,S/AS01 malaria vaccine in African settings. | Regional primary research |
|  | Galactionova, Bertram, Lauer and Tediosi ^12^ | To estimate the cost of vaccine introduction using routinely collected and publicly available data in Burkina Faso, Ghana, Kenya, Senegal, Tanzania, and Uganda. | Regional primary research |
|  | WHO ^9^ | To assess the malaria vaccine safety, impact (includes cost-effectiveness) and feasibility in the context of routine immunisation programmes. | Regional primary research |
|  | El-Houderi, Constantin, Castelnuovo and Sauboin ^13^ | To review published information on health system costs, health care resource use, and household costs for the management of malaria episodes in under-five children in sub-Saharan Africa | Regional systematic review |
|  | WHO ^14^ | To estimate malaria vaccine global supply, global demand and expected price per dose. | WHO global market study |
| **Health policy and programmatic considerations** | WHO ^9^ | To assess the malaria vaccine safety, impact (includes cost-effectiveness) and feasibility in the context of routine immunisation programmes. | Regional primary research |
|  | Hogan, Winskill and Ghani ^15^  Hogan, Winskill and Ghani ^15^ | To identify where vaccine introduction should be prioritised to maximise public health impact under a range of supply constraints using mathematical modelling in sub-Saharan Africa. | Regional primary research |
|  | WHO ^14^ | To estimate malaria vaccine global supply, global demand and expected price per dose. | WHO global market study |
| **Acceptability** | Ojakaa, Yamo, Collymore, Ba-Nguz and Bingham ^16^ | To explore perceptions of malaria and vaccines in Kenya | Local primary research |
|  | WHO ^9^ | To provide explanatory insight on the delivery and uptake of the RTS,S malaria vaccine in the pilot implementing counties in Kenya | Local primary research |
|  | Immurana, Boachie, Klu, Dalaba, Manyeh and Alhassan ^17^ | To examine the determinants of malaria vaccine awareness and willingness to uptake the vaccine for children in Ghana | Regional primary research |
|  | Dimala, Kika, Kadia and Blencowe ^18^ | To systematically review and summarize published studies addressing the challenges faced during the implementation phase of malaria vaccination programs and randomized trials conducted in sub-Saharan Africa. In addition, the review also sought to report proposed solutions to the challenges identified. | Regional systematic review |
| **Equity** | D'Souza and Nderitu ^19^ | Ethical considerations for introducing RTS,S/AS01 in  countries with moderate to high Plasmodium falciparum  malaria transmission | Global guidance on ethical considerations |

# **References**

1.	Kenya Malaria Indicator Survey 2020 (2020).

2.	Macharia PM, Giorgi E, Noor AM, et al. Spatio-temporal analysis of Plasmodium falciparum prevalence to understand the past and chart the future of malaria control in Kenya. *Malaria Journal*. 2018/09/26 2018;17(1):340. doi:10.1186/s12936-018-2489-9

3.	Alegana VA, Suiyanka L, Macharia PM, Ikahu-Muchangi G, Snow RW. Malaria micro-stratification using routine surveillance data in Western Kenya. *Malaria Journal*. 2021/01/07 2021;20(1):22. doi:10.1186/s12936-020-03529-6

4.	Alegana VA, Macharia PM, Muchiri S, et al. Plasmodium falciparum parasite prevalence in East Africa: Updating data for malaria stratification. *PLOS Glob Public Health*. Dec 7 2021;1(12):e0000014. doi:10.1371/journal.pgph.0000014

5.	Kamau A, Paton RS, Akech S, et al. Malaria hospitalisation in East Africa: age, phenotype and transmission intensity. *BMC Medicine*. 2022/01/27 2022;20(1):28. doi:10.1186/s12916-021-02224-w

6.	WHO. *World Malaria Report 2021*. 2021.

7.	WHO. *WHO Guidelines for malaria, 31 March 2022.* . 2022.

8.	RTSS Clinical Trials Partnership. Efficacy and safety of RTS,S/AS01 malaria vaccine with or without a booster dose in infants and children in Africa: final results of a phase 3, individually randomised, controlled trial. *Lancet*. Jul 4 2015;386(9988):31-45. doi:10.1016/s0140-6736(15)60721-8

9.	WHO. *Full Evidence Report on the RTS,S/AS01 Malaria Vaccine*. 2021. <https://cdn.who.int/media/docs/default-source/immunization/mvip/full-evidence-report-on-the-rtss-as01-malaria-vaccine-for-sage-mpag-(sept2021).pdf>

10.	WHO. *Malaria vaccine: WHO position paper – March 2022*. 2022.

11.	Penny MA, Verity R, Bever CA, et al. Public health impact and cost-effectiveness of the RTS,S/AS01 malaria vaccine: a systematic comparison of predictions from four mathematical models. *Lancet*. Jan 23 2016;387(10016):367-375. doi:10.1016/s0140-6736(15)00725-4

12.	Galactionova K, Bertram M, Lauer J, Tediosi F. Costing RTS,S introduction in Burkina Faso, Ghana, Kenya, Senegal, Tanzania, and Uganda: A generalizable approach drawing on publicly available data. *Vaccine*. Nov 27 2015;33(48):6710-8. doi:10.1016/j.vaccine.2015.10.079

13.	El-Houderi A, Constantin J, Castelnuovo E, Sauboin C. Economic and Resource Use Associated With Management of Malaria in Children Aged <5 Years in Sub-Saharan Africa: A Systematic Literature Review. *MDM Policy Pract*. Jul-Dec 2019;4(2):2381468319893986. doi:10.1177/2381468319893986

14.	WHO. *Global market study: malaria vaccine*. 2021. 2021. <https://apps.who.int/iris/handle/10665/350564>

15.	Hogan AB, Winskill P, Ghani AC. Estimated impact of RTS,S/AS01 malaria vaccine allocation strategies in sub-Saharan Africa: A modelling study. *PLoS Med*. Nov 2020;17(11):e1003377. doi:10.1371/journal.pmed.1003377

16.	Ojakaa D, Yamo E, Collymore Y, Ba-Nguz A, Bingham A. Perceptions of malaria and vaccines in Kenya. *Hum Vaccin*. Oct 2011;7(10):1096-9. doi:10.4161/hv.7.10.17496

17.	Immurana M, Boachie MK, Klu D, Dalaba MA, Manyeh AK, Alhassan RK. Determinants of willingness to accept child vaccination against malaria in Ghana. *Int J Health Plann Manage*. May 2022;37(3):1439-1453. doi:10.1002/hpm.3406

18.	Dimala CA, Kika BT, Kadia BM, Blencowe H. Current challenges and proposed solutions to the effective implementation of the RTS, S/AS01 Malaria Vaccine Program in sub-Saharan Africa: A systematic review. *PLoS One*. 2018;13(12):e0209744. doi:10.1371/journal.pone.0209744

19.	D'Souza J, Nderitu D. Ethical considerations for introducing RTS,S/AS01 in countries with moderate to high Plasmodium falciparum malaria transmission. *Lancet Glob Health*. Dec 2021;9(12):e1642-e1643. doi:10.1016/s2214-109x(21)00498-8
